# Extreme intrusive force affects the expression of c-Fos and matrix metallopeptidase 9 in human dental pulp tissues: Erratum

**DOI:** 10.1097/MD.0000000000026852

**Published:** 2021-08-06

In the article “Extreme intrusive force affects the expression of c-Fos and matrix metallopeptidase 9 in human dental pulp tissues”^[1]^ which appears in Volume 99, Issue 9 of *Medicine*, the authors are concerned that the images for part B in Figure 2 and 4 were too similar in the original version, which we selected for the negative control group. To avoid misunderstanding, Figure 4 has been updated with a new B.

Figure 4 has been replaced with:

**Figure:**
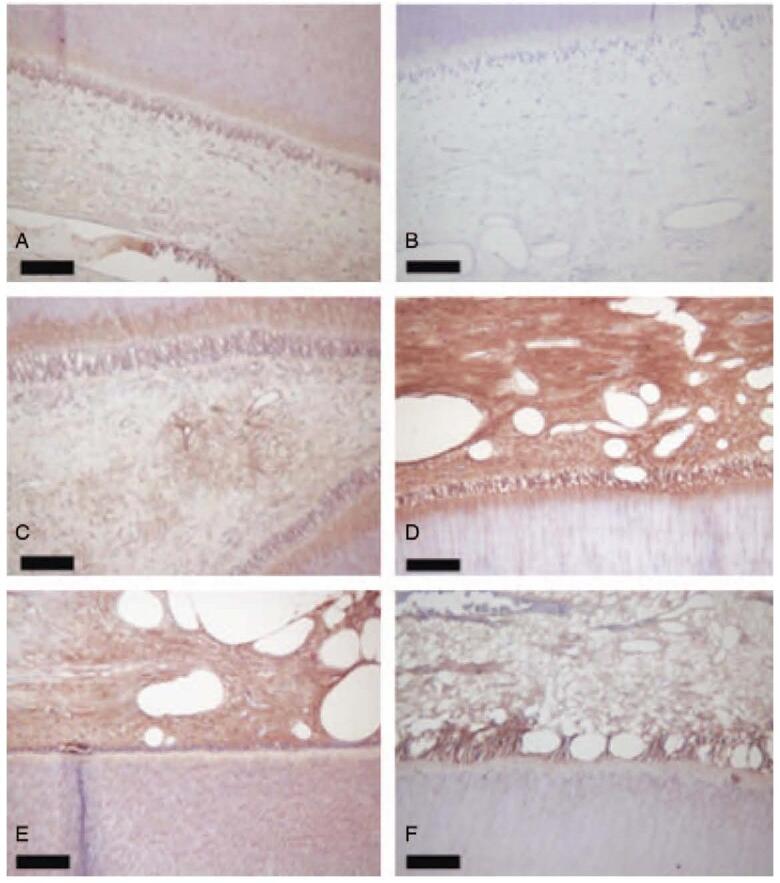


Figure 4. Typical images of immunohistochemical staining of MMP-9 in coronal dental pulp tissues. A: MMP-9 staining was weak in the cytoplasm of odontoblasts and dental pulp cells from patient group t = 0; B: MMP-9 staining was absent in negative control staining; C: MMP-9 staining was strong in the odontoblasts layer and underneath and around the blood vessels from patient group t = 1; D: MMP-9 staining was strong in the odontoblasts, dental pulp cells, and the blood vessels from patient group t = 4; E: MMP-9 staining was strong in the odontoblasts and perivascular vessels from patient group t = 8; F: MMP-9 staining was strong in the odontoblasts and the pulp cells from patient group t = 12. Scale bar: 20 mm.

The change does not affect the scientific results.
